# Remarks on Gude’s Pepto-Mangan

**Published:** 1898-04

**Authors:** T. V. Hubbard

**Affiliations:** Atlanta, Ga.


### REMARKS ON GUDE'S PEPTO-MANGAN.

By T. V. HUBBARD, M.D.,

ATLANTA, GA.

The result to be accomplished in the administration of any preparation of iron is primarily an increase in the number and quality of red blood corpuscles, and notably the hemoglobin, and secondarily an improvement in the general health which follows the increased oxygen-carrying power of the red cells. How to obtain this desirable end quickly and pleasantly with least inconvenience to the patient has been the constant aim of the physician. While we have numerous preparations of iron, they are practically all open to the same objection, that is, their astringent effect, though varying, of course, in degree. They most all produce more or less constipation, headache and derangement of the digestive organs. As a very small proportion of the inorganic iron is absorbed, it is probable that better results would be obtained in giving it in small doses, unless we admit the correctness of the theory of Bunge: that is, that in anemia there is frequently an excess of suphur or sulphureted hydrogen in the bowels, which unites with the medicinal iron administered, forming the sulphide of iron, and excreted with the feces, and thus allowing the organic iron of the food to be absorbed, and acting indirectly as a blood tonic. Though this theory may be true in part, it cannot be wholly so, because there is no evidence to show that food-iron is absorbed in preference to inorganic preparations when they are both in the intestines, and Stohman has cured anemia by giving iron hypodermically, and thus proven that inorganic iron does promote blood manufacture, and also disproving to a certain extent at least the correctness of Bunge's theory by showing that the inorganic iron does not necessarily act by uniting with the sulphur in the bowels. The truth probably is that the inorganic iron acts both ways to a certain extent, by being absorbed and furnishing material for the blood-making glands, and indirectly by uniting with the sulphur in the bowels, thus allowing absorption of all the food-iron.

In the administration of Pepto-mangan (which is an organic peptonate of iron and manganese) we have usually gotten more decided results and in a shorter time than with any other preparation of iron. Whether the superiority of this preparation is due to the special chemical form of the iron or to the manganese which exists in combination with it, or both, we are unable to say; but the practical results obtained from the administration of Pepto-mangan speak for themselves. We are aware of the fact, of course, that manganese itself exists in the blood in small quantities, and it is claimed by some that manganese possesses greater ozonizing power in the red blood corpuscles than iron; consequently the superior results obtained from this preparation over that of other preparations of iron is probably due to the manganese present, for the physician, in prescribing the ordinary preparation of iron, rarely ever combines manganese with it. Pepto-mangan is also very pleasant to the taste, neutral in reaction, does not injure the teeth, and is devoid of astringency, and is easily assimilated. It is claimed that this preparation of iron does not undergo any chemical change in the stomach before being absorbed, as does the inorganic salts of iron.

As an illustration of the prompt results obtained by this preparation, we will report a few cases in which it was used:

CASE 1.—Mrs. G., aged 28, six months in her second pregnancy, was very much emaciated, anemic, and unable to exercise much for want of muscular strength. She also suffered much loss of appetite, was very nervous at night, so much so that she was unable to sleep, and had the general appearance of a profound anemia. After taking Pepto-mangan for two or three weeks she was practically restored to her normal condition of health; her color returned to lips and cheeks, her nervous symptoms disappeared, and she was able to sleep comfortably.

CASE 2.—Boy, aged 8; had the mumps, ulcerated throat, accompanied by a general glandular enlargement in the neck; was very anemic and somewhat of a scrofulous tendency. After the acute symptoms of mumps had subsided, Pepto-mangan was administered in drachm doses three times a day after meals. The throat symptoms improved, the enlarged glands disappeared, color was restored, and there was general improvement in the nutrition after two weeks' administration.

Numerous other cases could be mentioned in this connection confirming the above, but we think the beneficial results to be obtained from Gude's Pepto-mangan are too well known by the medical fraternity to need any further comment.

---

### MAMMARY CANCER—PROGNOSIS.

The prognosis after operation for mammary cancer is thus summed up by Dr. Bennett May in his lectures, just published: "So far as is at present known, our only hope for advancement lies in the direction of more thorough and more early operation. The limit of what is possible in the former direction will soon be, if it is not already reached. The result must carry conviction that we may hopefully anticipate a real cure in at least 30 or 40, or some would say 50, per cent. of our cases. I would not like to place 30 as a limit, but it is with somewhat chastened hope that I look for anything beyond. Longer observation is required, particularly as to the future development of those cases who pass the three-year limit. Even now, however, the operation in too many cases is not practiced to the best advantage, and is not used for all it is worth. Certainly some of the disrepute and prejudice which have surrounded it may fairly be ascribed to the incomplete and inadequate manner in which it is too often done by men who have had no proper surgical training, and whose ill results serve to injure the cause as a whole, and to reflect prejudicially on the work of others. The fact is it has been every one's operation because it is thought to be easy, but now that surgery is specialized to such an extent these happy-go-lucky methods should be abolished in this as in other branches of surgery."—*Am. Jour. Surg. and Gyn.*

